# Stretches of alternating pyrimidine/purines and purines are respectively linked with pathogenicity and growth temperature in prokaryotes

**DOI:** 10.1186/1471-2164-10-346

**Published:** 2009-07-31

**Authors:** Jon Bohlin, Simon P Hardy, David W Ussery

**Affiliations:** 1 Norwegian School of Veterinary Science, P.O. Box 8146 Dep., N-0033 Oslo, Norway; 2 National Veterinary Institute, Pb. 750 Sentrm, N-0106 Oslo, Norway; 3 Center for Biological Sequence Analysis, Department of Systems Biology, Technical University of Denmark, DK-2800 Lyngby, Denmark

## Abstract

**Background:**

The genomic fractions of purine (RR) and alternating pyrimidine/purine (YR) stretches of 10 base pairs or more, have been linked to genomic AT content, the formation of different DNA helices, strand-biased gene distribution, DNA structure, and more. Although some of these factors are a consequence of the chemical properties of purines and pyrimidines, a thorough statistical examination of the distributions of YR/RR stretches in sequenced prokaryotic chromosomes has to the best of our knowledge, not been undertaken. The aim of this study is to expand upon previous research by using regression analysis to investigate how AT content, habitat, growth temperature, pathogenicity, phyla, oxygen requirement and halotolerance correlated with the distribution of RR and YR stretches in prokaryotes.

**Results:**

Our results indicate that RR and YR-stretches are differently distributed in prokaryotic phyla. RR stretches are overrepresented in all phyla except for the Actinobacteria and β-Proteobacteria. In contrast, YR tracts are underrepresented in all phyla except for the β-Proteobacterial group. YR-stretches are associated with phylum, pathogenicity and habitat, whilst RR-tracts are associated with phylum, AT content, oxygen requirement, growth temperature and halotolerance. All associations described were statistically significant with *p < 0.001*.

**Conclusion:**

Analysis of chromosomal distributions of RR/YR sequences in prokaryotes reveals a set of associations with environmental factors not observed with mono- and oligonucleotide frequencies. This implies that important information can be found in the distribution of RR/YR stretches that is more difficult to obtain from genomic mono- and oligonucleotide frequencies. The association between pathogenicity and fractions of YR stretches is assumed to be linked to recombination and horizontal transfer.

## Background

Frequencies of RR and YR stretches of 10 bp or more have been associated with several genomic and DNA structural features [1,2]. For instance, purine and pyrimidine patterns have been found to be better conserved than base composition in all domains of life [3]. Short runs of the purine adenine (A) have been linked to curved DNA sequences [4,5], and purine asymmetry is associated with strand-biased gene distribution [6]. Runs of YR stretches tend to form Z-DNA helices in GC-rich sequences, and some purine tracts are associated with A-DNA helices [1]. DNA helices are in general linked with both sequence patterns and environmental conditions [1]. A- and B-DNA type helices appear to be more common in genomic DNA [1]. However, local and less frequent variants known as C-, D- and T-DNA helices can also occur [5]. The left-handed Z-DNA helix is found less frequently in prokaryotes than in eukaryotes and appears to be unstable in bacteria [7].

While RR and YR stretches are short-range correlated in archaea and bacteria, their distribution in eukaryotes is more complex [1]. In this work, we focus on prokaryotes. The distributions of RR/YR tracts in prokaryotes have been described previously [1,4], but many issues have not been resolved. The large number of sequenced genomes available enabled us to search for possible factors associated with the distribution of RR/YR stretches. This was carried out by examining RR/YR tracts containing 10 bp and more in 546 chromosomes from 494 genomes. To reduce bias, similar species and species with many sequenced strands were removed from the original dataset consisting of 865 prokaryotic DNA sequences. One turn of the DNA helix is in the range of 10 bp for the most common helices [1,5] and this guided our choice for the RR/YR sequence length. Regression analysis was subsequently carried out to compare frequencies of genomic RR and YR-stretches with genome size, AT content, phyla, oxygen requirement, habitat, growth temperature, pathogenicity and halotolerance.

## Results

**Figure 1:**
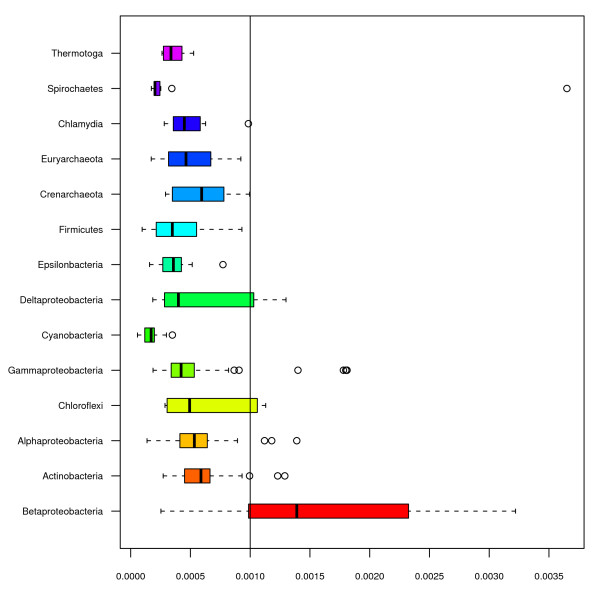
**The graph depicts the genomic distribution of alternating pyrimidine/purine (YR) stretches of 10 bp or more in prokaryotic phyla**. The expected fraction is 0.001 (0.1%). It can be seen that the β-Proteobacterial group has more than the expected fractions of YR-stretches, while all other groups have, on average, less than expected.

To measure possible factors influencing the distribution of RR and YR stretches in prokaryotes, two regression models were fitted. For both models, AT content, phylum, oxygen requirement, habitat, temperature, pathogenicity and halotolerance were tested as predictors. The tested predictors not found significant were removed from the model. For the YR model, phyla, habitat and pathogenicity were found significant (*p < 0.001*). Of the included predictors it can be seen from Table 1 that phyla added the largest improvement to the model, followed respectively by AT content and pathogenicity. The model describing the distribution of YR stretches obtained a coefficient of determination *R*^2 ^= *0.53*. According to the simple model explained in the materials and methods section, the distributions of YR stretches were found to be underrepresented in both archaea and bacteria (see Figure 1), which is in accordance with previous work [1]. β-Proteobacteria was the only group found to have, on average, an overrepresentation of YR stretches compared to what was expected, *i.e*. 0.1%. To further examine the relationship between YR stretches and pathogenicity, a binomial regression model was fitted with the dichotomous factor pathogenicity as response. The factors found significant (*p < 0.001*) were RY-stretches and habitat. To remove bias, clustering with respect to phylum was included which resulted in a decreased Z-score from 5.4 to 4.11 (*p < 0.001*).

**Table 1:** YR- stretches regression model

AIC	Factor	*R*^2^	AIC Difference
1134	Constant		
1010	AT content	0.2	124
782	Phyla	0.5	228
755	Habitat	0.52	27
743	Pathogenicity	0.53	12

Results from forward fitting a regression model with genomic fractions of YR stretches from 546 chromosomes as response. Factors were added successively and those not found significant (*p *< 0.001) were discarded from the model.

**Figure 2:**
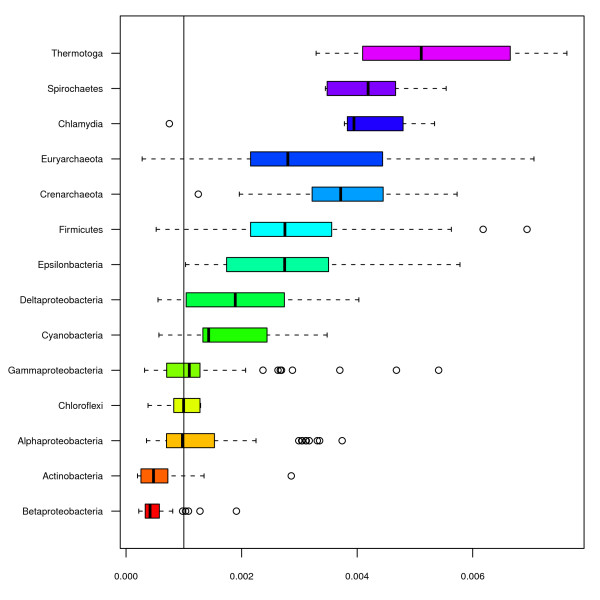
**The box-plot shows the distribution of genomic purine stretches consisting of 10 bp or more in prokaryotic phyla**. The expected genomic fraction of RR-stretches is 0.001 (0.1%). The Actinobacterial and β-Proteobacterial groups were the only ones found to be underrepresented in terms of genomic RR-stretches.

In Table 2 it can be seen that a considerably better model was obtained for the distribution of RR stretches in terms of 'coefficient of determination' (*R*^2^). Phyla was found to be the most important factor followed respectively by AT content, temperature, oxygen requirement, halotolerance and habitat. Our findings indicated that RR stretches were in general overrepresented (See Figure 2) which is also in agreement with previous work [4]. Actinobacteria and β-Proteobacteria were the only phyla with fewer RR stretches, on average, than expected. α-Proteobacteria, γ-Proteobacteria and Bacteroidetes/Chlorobi all contained the expected amount of RR stretches.

**Table 2:** RR- stretches regression model

AIC	Factor	*R*^2^	AIC Difference
1414	Constant		
1043	AT content	0.49	371
725	Phyla	0.73	318
694	Oxygen requirement	0.74	31
690	Habitat	0.75	4
630	Growth temperature	0.77	60
608	Halotolerance	0.78	22

Results from forward fitting a regression model with genomic fractions of RR stretches from 546 chromosomes as response. Factors were added successively and those not found significant (*p *< 0.001) were discarded from the model.

It should be noted that models based on the reverse compliments of the RR and YR-models, *i.e*. YY and RY-stretches, produced similar results. It is assumed that this is due to Chargaff's parity laws,*i.e*. purine and pyrimidine levels are the same throughout chromosomes, but may be differently distributed along each strand.

**Figure 3:**
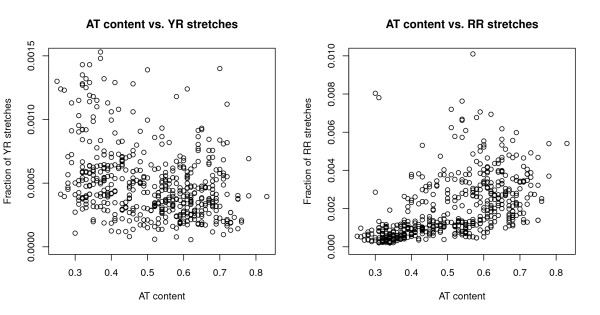
**The graph on the left shows genomic AT content (horizontal axis) versus the genomic fractions of alternating pyrimidine/purine stretches of 10 bp (vertical axis)**. The graph on the right shows a similar plot, but for the fraction of purine stretches (10 bp). With all outliers removed it can be seen from the left graph that there is low linear correlation between genomic fractions of YR stretches and AT content (*R*^2 ^= *0.12, p < 0.001*), while higher correlation persists between purine stretches and AT content (*R*^2 ^= *0.28, p < 0.001*).

In Figure 3, it can be observed that the genomic fraction of RR and YR stretches were differently affected by genomic AT content. While the squared Pearson correlation coefficient between genomic YR stretches and AT content was *R*^2 ^= *0.12*, the corresponding squared correlation coefficient was *R*^2 ^= *0.28 *for genomic RR stretches.

**Figure 4:**
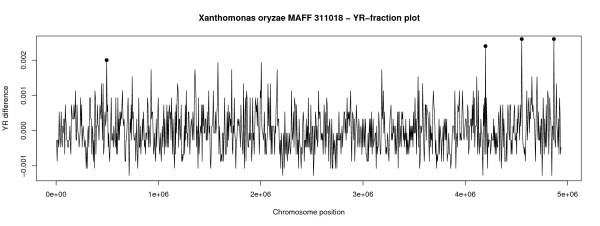
**The graph shows a genomic profile of the plant-pathogen *Xanthomonas oryzae *MAFF 311018 based on the computed differences between the genomic fractions of YR-stretches and a non-overlapping sliding windows of 5 kbp**. The peeks having a difference above 0.002 (0.02%) were marked and the corresponding genetic regions were BLASTed against Genbank. The hits retrieved from BLAST indicated that all regions were linked to mobile genetic elements associated with recombination and horizontal transfer.

To examine possible relations between overrepresentation of YR stretches and pathogenicity, we analyzed the difference between the frequencies of YR stretches in a sliding window and the genome of *Xanthonomonas oryzae *MAFF 311018 (Figure 4). All regions with a difference above 0.002 (0.2%) were extracted and blasted. The regions found in positions 4190001–4199960, 4545001–4559999 and 4860001–4869999 included transposons and transposase genes. Transposons are mobile genetic elements associated with recombination events and horiztonal transfer [8]. The region 1577001–1583000 contained a RND superfamily protein. This protein is commonly associated with antibiotic resistance [9].

## Discussion

The results above represents a continuation of earlier work [1,4], but limited to prokaryotic genomes. Previously [1], it was demonstrated that the distribution of RR and YR stretches in eukaryotes were very different to prokaryotes. That is, the distribution of YR and RR stretches in eukaryotic genomes deviate strongly from the Markov-based, short-range correlation model used for prokaryotes. The constraints responsible for the different distributions of RR and YR stretches between prokaryotic and eukaryotic organisms are not known, but may possibly be attributed to the non-linear, multi-scaled and highly fractal organization of nucleotides in eukaryotic genomes not observed in prokaryotes [10].

Analyses of the distribution of RR and YR stretches in prokaryotic chromosomes (figures 1 &2) reveal that while YR stretches of 10 bp tend to be underrepresented according to what is expected, RR stretches are to a large extent overrepresented. For YR stretches this is true for all phyla except β-Proteobacteria of which the GC-rich *Burkholderia *genus is found to have a larger fraction YR stretches than any other genus (see Figure 1). As has been noted earlier, YR stretches may form Z-DNA in GC-rich sequences, and Z-DNA is highly unstable in bacteria [7]. In general, YR stretches tend to be associated with genome arrangement and recombination [11,12]. In mammals, Z-DNA formation has been found to generate large genetic alterations possibly associated with certain types of cancer [7]. The observation that pathogenicity was a significant factor (*p < 0.001*) describing YR stretches in bacterial genomes was therefore of considerable interest.

The *Burkholderia *species are also known to contain many CG repeats which are, in general, associated with Z-DNA formation [1,13]. Horizontal transfers and frequent DNA exchange is also common within the *Burkholderia *genus [14]. The significance of the pathogenicity factor reduced to *t~2.0 *(*p < 0.05*) when the entire *Burkholderia *genus, consisting of 32 chromosomes, and the extreme outlier *Treponema pallidum *were removed from the dataset. In contrast, when the fraction of RR stretches was exchanged as response for the fraction of YR-stretches in the same model for the same dataset, the resulting significance was *t = -0.4*, *p~0.7*. The reduced dataset contained 194 pathogenic and 318 non-pathogenic chromosomes, while the main dataset included 222 pathogenic and 324 non-pathogenic chromosomes.

The finding that alternating pyrimidine/purine stretches of 10 bp or more are significantly associated with pathogenicity may indicate that YR tracts are positively correlated with genomic regions in bacteria that are susceptible to recombination or horizontal gene transfers resulting in the acquisition of pathogenicity islands. The fact that YR-stretches are underrepresented in prokaryotic genomes may suggest a counter selection of unstable regions. This is in stark contrast to what is observed in many eukaryotic organisms [1].

Purine stretches are overrepresented in all phyla except for the γ-Proteobacteria, Bacteroidetes/Chlorobi and α-Proteobacteria groups. Actinobacteria and β-Proteobacteria are the only groups found to have a lower than expected fraction of purine stretches. From figures 1 and 2 it can be seen that fractions of RR stretches were most diversely distributed in archaea, while β-Proteobacteria had the most varied distributions of YR stretches. The over- and underrepresentation of RR and YR stretches is also presumed to be influenced by DNA helix preference [1].

Both models revealed several important factors associated with the respective distribution of RR and YR stretches. The best model, in terms of *R*^2^, was obtained for the distribution of RR stretches. This implies that there may be different factors shaping the distributions of RR and YR stretches in bacterial genomes. This is supported by the regression models which found different factors significant. While AT content, extreme halotolerance, oxygen requirement, and growth temperature were significant factors in the RR based regression model, habitat and pathogenicity were found to be significant in the YR-model. The phyla factor was significantly associated with both RR and YR based regression models.

The model explaining RR stretches found oxygen requirement and growth temperature as important and significant factors (*p < 0.001*). GC content has been associated with oxygen requirement in prokaryotes [15]. A slight, but significant (*p < 0.001*), improvement was obtained by adding the oxygen requirement factor to the RR-based regression model, but the addition of the growth temperature factor improved the model considerably. Why thermophilicity and halotolerance is linked with the distributions of purine tracts is not known, but RR-stretches appear to be more stable compared to YR-stretches [4]. Genomic GC content resists any linear association with growth temperature (*p > 0.5 *from our data, using a transformed regression model) [16,17]. However, the GC content of RNA genes has been found to correlate with growth temperature [18,19], and purine tracts are overrepresented in mRNA sequences of thermophilic prokaryotes [2]. The association between RR stretches and growth temperature was very clear compared to that of genomic AT content and growth temperature.

That AT content is an important factor for oligonucleotide frequencies has been noted previously [20]. To what extent AT content affects the distribution of RR stretches in prokaryotes has, to the best of our knowledge, not been accurately described for prokaryotes (see Figure 3). It has been observed that many bacteria from the AT-rich Firmicutes group tend to prefer purines on the leading strand [1]. Genomes having an overrepresentation of purine stretches on the leading strand have additionally been found to carry a PolC proof-reading enzyme [21]. It is therefore also possible that an excessive distribution of purine stretches is associated with the polC gene. More data is needed however, before this can be examined further.

All regression models suffer from the effect of co-linearity. That is, several predictor variables overlap to some extent in terms of explaining the variance in the model. For instance, AT content has been found to correlate with genome size [22] and some co-linearity is also assumed between phyla and AT content. Therefore, the exact influence of the different predictors in the models can not be precisely stated and the models presented have the primary function of identifying significant influences as a starting point for further analysis.

Overrepresentation of YR stretches in *Xanthonomonas oryzae *MAFF 311018 is found to be associated with transposons and a 'RND complex' [9], both of which are connected to mobile genetic elements and horizontal transfer. The RND complex is also found in many other bacteria, and the associated outer membrane protein found in the *Xanthonomonas oryzae *MAFF 311018 genome is presumably promiscuous [23]. Thus, preliminary analysis may indicate that YR-stretches may play some role in the life of mobile genetic elements and that this may be the link we found to pathogenicity.

## Conclusion

The regression models varied in terms of goodness of fit/coefficient of determination (*R*^2^). The genomic distributions of YR stretches were not as adequately described by the regression model as the RR-stretches. This indicates that there are additional factors that remain to be identified for the YR-based regression model. The relatively high coefficient of determination obtained for both RR and YR-based regression models was surprising. It was of great interest to note that temperature was such an important factor in the RR-model, and that pathogenicity was significant in the YR-model.

We assume that the correlation between pathogenicity and YR-stretches is due to an increased tendency of Z-DNA formation in areas overrepresented with YR-stretches. Z-DNA formation has been associated with recombination and genetic rearrangements [12], and it may therefore be a higher probability of horizontal transfers, recombination and gene uptake in such areas. RR-stretches are known to be more stable than YR-stretches [1] and this is presumably the reason they are overrepresented in the genomes of thermo- and halophilic prokaryotes.

## Methods

The genomic DNA sequences and information used in the models as factors were downloaded from the NCBI database [24]. Only one strand from each species was included, and all plasmids were excluded. The total number of chromosomes was 546 representing 494 genomes from 22 different phyla. A computer program was written to count overlapping 10 bp RR/YR stretches using a 2^10 ^entry hash table containing the maximal number of occurring stretches. A variant of this program was made to find the difference between non-overlapping sliding windows and genome based frequencies of YR stretches. The program was used to examine overrepresented YR-stretches in *Xanthonomonas oryzae *MAFF 311018. The *X. oryzae *genome was chosen since it is a known plant pathogen [25] and it contains a relatively large fraction of YR stretches. Genomic regions with an YR-difference above 0.002 (0.2%) were BLASTed against Genbank [24,26]. The sliding window size was set to 5 kbp. The programs are available by request from the corresponding author, and the dataset used is included as Additional file 1.

Chargaff's parity rule [27] states that the ratio of purines and pyrimidines is approximately equal in all genomic DNA sequences. We therefore expected that the frequency of both RR and YR stretches consisting of 10 nucleotides to be (1/2)^10 ^or about ~0.001. In other words, it is expected that all possible combinations of 10 bp purine and pyrimidine stretches occur with 0.1% probability. This simple background model assumes that each nucleotide is independent of its nearest neighbor.

The models were created using regression analysis with RR and YR frequencies as response variables and genome size, AT content, phyla, growth temperature, oxygen requirement, habitat, pathogenicity and halotolerance as predictors. Each response variable was log-transformed to optimize the fitting of residuals to the normal distribution. The following equation was obtained for the modeling of RR-stretches:



while the following equation was used to examine YR-stretches:



Oxygen, habitat, temperature, phyla and halotolerance were categorical factors. Oxygen requirement consisted of the factors: aerobic, anaerobic and facultative. The habitat factor consisted of the following categories: host-associated, specialized, terrestrial, multiple, and aquatic. Temperature was a factor with these given categories: psychrophilic, mesophilic and thermophilic. Halotolerance included the factors: non-halophilic, mesophilic, halophilic and extreme halophilic. Genome size was excluded from both models since it was not found to be significant.

To verify how the different predictors affected the pathogenicity factor, a binomial regression model was fitted with the dichotomous pathogenicity factor as the response and AT content, RR-streches, YR-stretches and habitat as predictors. The factors representing AT content, RR and YR stretches were numeric, while habitat was a categorical variable. Clustering was performed with respect to phyla to correct for intra-correlations within each phylum. The following model was fitted:



Statistical analyses were performed with the program R [28].

## Abbreviations

YR-stretches: Alternating pyrimidine/purine stretches of more than 10 bp; RR-stretches: purine stretches of more than 10 bp

## Authors' contributions

JB wrote the manuscript, carried out statistical analyses and wrote the computer programs. SH critically drafted and revised the manuscript, DWU conceived of the study, performed analyses and critically drafted and revised the manuscript. All authors read and approved the final manuscript.

## Supplementary Material

Additional file 1 An Excel file containing the dataset used to generate the results in the article. Click here for file
